# Sensors for Biomass Monitoring in Vegetated Green Infrastructure: A Review

**DOI:** 10.3390/s23146404

**Published:** 2023-07-14

**Authors:** Farhad Jalilian, Caterina Valeo, Angus Chu, Rustom Bhiladvala

**Affiliations:** 1 Mechanical Engineering, University of Victoria, Victoria, BC V8W 2Y2, Canada; farhadj@uvic.ca (F.J.); rustomb@uvic.ca (R.B.); 2 Civil Engineering, Schulich School of Engineering, University of Calgary, Calgary, AB T2N 1N4, Canada; achu@ucalgary.ca

**Keywords:** stormwater pollution, bioretention, biological remediation, bacteria biomass sensing

## Abstract

Bioretention cells, or rain gardens, can effectively reduce many contaminants in polluted stormwater through phytoremediation and bioremediation. The vegetated soil structure develops bacterial communities both within the soil and around the vegetation roots that play a significant role in the bioremediative process. Prediction of a bioretention cell’s performance and efficacy is essential to the design process, operation, and maintenance throughout the design life of the cell. One of the key hurdles to these important issues and, therefore, to appropriate designs, is the lack of effective and inexpensive devices for monitoring and quantitatively assessing this bioremediative process in the field. This research reviews the available technologies for biomass monitoring and assesses their potential for quantifying bioremediative processes in rain gardens. The methods are discussed based on accuracy and calibration requirements, potential for use in situ, in real-time, and for characterizing biofilm formation in media that undergoes large fluctuations in nutrient supply. The methods discussed are microscopical, piezoelectric, fiber-optic, thermometric, and electrochemical. Microscopical methods are precluded from field use but would be essential to the calibration and verification of any field-based sensor. Piezoelectric, fiber-optic, thermometric, and some of the electrochemical-based methods reviewed come with limitations by way of support mechanisms or insufficient detection limits. The impedance-based electrochemical method shows the most promise for applications in rain gardens, and it is supported by microscopical methods for calibration and validation.

## 1. Introduction

Stormwater is the excessive amount of rainfall or melted snow that does not sink into the ground and flows over the landscape surface to eventually find its way to a receiving water body. Impervious surfaces, which are prevalent in urban areas, generate large amounts of stormwater because they are compact surfaces with low permeability (such as roads and parking lots) and do not allow the rainfall to penetrate the subsurface [1]. Stormwater flow captures and conveys contaminants and pollutants left on the surface between rain events. Increasing urbanization leads to increasing impervious surface area [2], and a wide variety of undesired contaminants are captured by stormwater in urban areas. According to Lefevre et al. [3], pesticides, pathogens, Total Suspended Solids (TSS), heavy metals, dissolved solids, petroleum hydrocarbons, and organic chemicals can be present in urban stormwater runoff. Subsequently, urban storm water runoff has resulted in the degradation of the quality of receiving water bodies and, in turn, adversely affected human and ecological health.

Water quality is a significant component of stormwater management. Stormwater management involves flood prevention and increasing the quality of stormwater runoff before it discharges into receiving waters. Modern stormwater management includes Sustainable Urban Designs (SUDs) and Low-Impact Development (LID), which integrates land-use planning and engineering design to manage storm water runoff [1]. Generally speaking, conservation of natural sites and utilizing them to increase water quality are the main goals of LID, and they include retention ponds, green roofs [3] bioswales, permeable pavements, constructed wetlands, and bioretention cells, also known as rain gardens [2].

**Figure 1:**
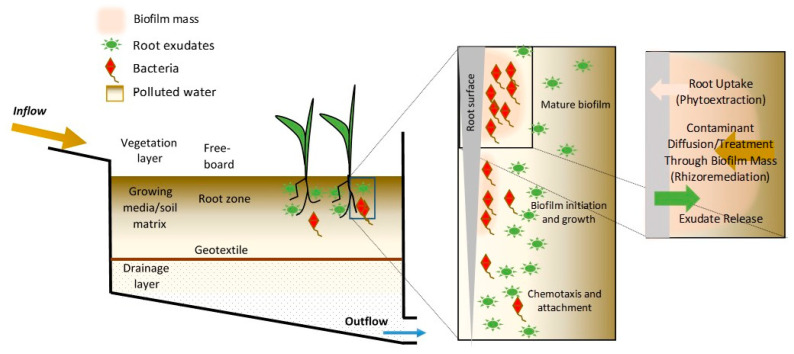
Schematic of bioretention cell showing some of the possible remediating processes. The freeboard is designed to capture specific design volumes and peaks and permit timed percolation to lower layer(s). The vegetation above the surface, the root zone, the lower sections of the growing media, and the drainage layer (if it exists) all contribute to the treatment of contaminants. Biofilm growth processes are shown near a root section with contaminate treatment through biofilm (rhizoremediation) and uptake by the roots.

Bioretention cells are a popular form of LID, particularly for its stormwater quality treatment capacity. Also termed biofilters, they are effectively biological media filters that improve water quality by temporarily storing large stormwater volumes [4,5]. Stormwater is directed into the bioretention cell, which is a shallow vegetated depression that has an engineered soil media [3]. Bioretention cells improve water and urban runoff quality via various physical, chemical, and biological processes that take place as water passes through the filtration media [2]. Figure 1 shows a schematic of a typical design for a bioretention cell. Once the stormwater begins percolating through the soil media, the toxicity of the runoff is decreased by capturing various pollutants [6]. The mulch layer provides an additional filtration barrier. Mulch and peat moss are often used in bioretention systems because of their high sorption capacity [7]. After the infiltration of stormwater through the cell is completed, the treated water can either trickle into the surrounding geologic environment or can be piped to other locations [2].

In general, bioretention cells can be used for a wide range of applications, from high infiltration to pretreatment of urban runoff [8]. The biological processes of water and soil quality improvement and contaminant removal that take place in bioretention cells are simply referred to as bioremediation. Bioremediation is a waste management method that effectively uses living cells to remove or neutralize pollutants. It can also be defined as the engineered utilization of vegetated depressions (as in bioretention cells) to extract, remove, sequester, degrade, and neutralize the pollutants from surface waters, groundwater, and soil [9].

### Bioremediation in a Rain Garden through Biofilm

The process of bioremediation in bioretention cells that uses specific types of plants for contaminant removal is called phytoremediation. This can be further classified into where the removal is conducted—either through, or at the roots, or further up the plant matrix. Scientists have identified more than 400 plant species with the potential for direct soil and water bioremediation [10]. However, not just the plants themselves, but also the microbial communities that exist in the vicinity of the roots —known as rhizospheric bacteria or rhizobacteria—are effective in removing pollutants. Plant roots release exudates that are nutrient-rich and play a crucial role in healthy biofilm growth [11,12] (see Figure 1). This bioremediation process using the plant-root-associated bacteria is called rhizoremediation and is an indirect bioremediation that is executed by the microbial processes in the rhizosphere. It is thought that when bacterial cells form a community, they become much more effective in bioremediation [13].

Among various species of microorganisms, certain types are capable of forming aggregates of bacterial cells, called biofilms. The key initiation mechanism for the formation of biofilms on surfaces such as plant roots is chemotaxis, which allows single planktonic bacterial cells to form functional glue-like biofilms [14]. Biofilms assist with the bioremediation not just by solubilizing and degrading contaminants but also by improving the health and growth of their host plants [13,15]. They are responsible for a considerable portion of the water quality improvements and enhance the efficacy of contaminant removal. Rhizobacteria are shown to reduce the detrimental impact of toxic metals on the growth and health of the host plants in highly heavy-metal-contaminated sites [16]. They can also decontaminate the soil from xenobiotic compounds that are recalcitrant and carcinogenic even when present at low concentrations [17]. In many industries, the formation of biofilms can lead to an increased risk of biofouling, corrosion, and infection [18,19,20], but in this context, biofilm formation and growth is part of effective wastewater and stormwater treatment. While numerous bioremediation processes are possible in a bioretention cell, including phytoextraction and rhizoremediation (see Figure 1), a primary pollutant of interest for stormwater managers that is remediated through a biofilm is nitrogen and related compounds. Microbial denitrification describes how nitrate (NO_3_^−^) or nitrite (NO_2_^−^) is converted to nitric oxide (NO), nitrous oxide (N_2_O), or nitrogen gas. More than 50 genera of bacteria have been identified as being capable of denitrification, but in general, the bacteria have one (or both) of two types of nitrate reductase, Nar and Nap [21]. Nitrifying bacteria such as those in the bacterial genus *Nitrosomonas* use ammonia nitrogen (NH_3_) by converting it to nitrite in the nitrifying process. The genus *Nitrobactor* then converts the nitrite to nitrate in the two-stage process called nitrification [22]. Nitrifying bacteria contain denitrification genes such as nirS, nirK, and nosZ [23]. Numerous other species leading to the bioremediation of contaminants such as hydrocarbons and pesticides have been studied and documented [24]. However, the community of bacteria in most functioning full-scale bioretention cells is highly diverse [23].

Biofilm can be formed as result of the aggregation of millions or single species of organic/inorganic compounds that can range from decomposing materials to microorganisms [25,26]. According to Vlamakis et al. [27], biofilms are assemblages of microorganisms that are surrounded by an Extracellular Polymeric Matrix (EPM) adhered to a surface. The attachment of freely available bacterial cells to an inert surface such as a plant root is the turning point where the planktonic life of bacteria turns into biofilm mode [28]. The biofilm life cycle can be divided into five different phases: the initial attachment of organisms to a surface (enhanced through root surface roughness [29]); followed by quorum sensing by the organisms [30,31], leading to EPM formation; maturation, in which cells attached to the surface start to divide and proliferate; a stationary phase, in which the growth stops; and finally, death, where cells detach and leave the colony [32]. Since nutrients are required for biofilm to grow and since waste removal is ongoing, bacterial growth potential is prone to be limited depending on biofilm structure and hydrodynamics, as well as nutrient limitations [31]. This means that rhizoremediation in a rain garden is affected by characteristics of the stormwater, the growing media, the vegetation, the rain garden’s physical characteristic affecting the hydrodynamics, and the climate.

Stormwater, unlike wastewater, is intermittent, with low concentrations of contaminants as compared to wastewater. The contaminants are nutrients for the rhizobacteria, which can undergo drastic fluctuations between rain events. The health, growth, and extent of the vegetation are certainly functions of the retention capacity of the soil and its organic content. These are most often predetermined by local conditions (indigenous vegetation, soil supply, costs, and climate) but are designed to promote healthy growth in the local environment. Bioretention cell function in cold weather is continually being researched [2], but rhizoremediation is expected to be fully functional during the plant growing season. For these reasons, any type of real-time monitoring of biomass activity from rhizoremediation (and, potentially, all other forms of bacterial activity in the root zone) in situ would likely have to occur when the biofilm reached full maturation and was at maximum extent in the vegetation growing season and after the rainy season.

Depending on the physical, chemical, and biological conditions of the environment of interest, methods of studying biofilm differ from one another in the sense that the monitoring method used in one environment or application may not be as useful when utilized in a different application [33]. Despite the research and availability of biosensors allowing for the real-time monitoring proposed in the literature [34], monitoring biofilm in real-time and in situ remains a challenge [35]. Rodriguez-Mozaz et al. [36] assert that in spite of past improvements in biosensor technology, there still exists the challenge of developing a more reliable device. To the authors’ knowledge, no practical, affordable real-time sensor for monitoring biofilm activity in a rain garden in situ exists. The objective of this work is to review the most popular and successful methods currently available for monitoring beneficial biofilms. The advantages and shortcomings of each method will be discussed for their potential use in a practical, real-time, in situ sensor of biofilm activity in a rain garden. Recommendations for just such a sensor are provided. This can aid research into viable in situ sensors for monitoring bacterial biofilm and provide an estimation of biomass availability in the context of water quality improvement from bioretention cells treating stormwater. Such a tool could be used by municipalities to determine if their rain gardens are functioning for remediation of water quality and to what degree, whether maintenance is required, or whether design changes are required to remain sustainable.

## 2. Review of Potential Methods for Sensing Biofilm Activity in a Rain Garden

All biofilm monitoring methods function on the basis of a response signal acquired from the biofilm. These signals are a result of energy transfer, light scattering, heat transfer, acoustic waves, electrical fields or electrical currents, and mechanical signals. The process of monitoring involves transmittance of input signals, modification of the input signals by the biofilm and its host environment, and detection of the output signals by the sensor. The biofilm leaves a characteristic footprint in the modified signal (output signal) [33]. Funari and Shen [35] provide a general overview of tools that have been used to investigate biofilm properties or their responses/metabolisms, as well as tools that use the biofilm as the actual sensing transducer to indicate other information. The focus of this review and discussion is on tools that provide measured data of biofilm formation and activity in situ, leading to stormwater treatment in a living rain garden.

### 2.1. Microscopical Methods for Measuring Biofilms

Conventional methods of studying biofilms involve the enumeration and morphological observation of the microorganisms using microscopic techniques such as light and epifluorescence microscopy, as well as confocal laser scanning microscopy (CLSM) and scanning electron microscopy (SEM). Light microscopy and epifluorescence techniques are suitable for biofilms with a thickness of less than 3–4 µm because the multilayered biofilm structures scatter the light emitted at the sample and disrupt direct observation. The dye 4′,6-diamidino-2-phenylindole (DAPI) is one of the few staining dyes that has been used widely by researchers for biofilm studies. DAPI binds to the double-stranded regions in the DNA of the cells and makes the visualization of dead and live adherent cells possible. Grohmann and Vaishampayan [37] reported great success in visualizing enamel biofilms when using DAPI staining combined with the fluorescence in situ hybridization (FISH) method. FISH, which utilizes rRNA-targeted probes for visualization, is the most common method of identifying the different microbes in a community of biofilms. The main reason why FISH is of high popularity among biofilm researchers is that it allows for the differentiation of the cells [38]. The FISH method’s capability can be largely enhanced when combined with confocal laser scanning microscopy (CLSM). CLSM, a subcategory of laser scanning microscopy (LSM), and allows for three-dimensional imaging of microbial structures. The main breakthrough that CLSM has brought to the field is that the biofilm samples can be imaged without fixation and while contained in their fully hydrated state in situ [39]. Point illumination and the elimination of out-of-focus light allows CLSM to trump the shortcomings of the conventional light microscopy, which is suitable for in-plane bacterial monitoring [40]. Despite the tremendous success of CLSM-FISH in the whole-cell monitoring of biofilms, the accurate enumeration of a bacterial population remains a challenge that requires the implementation of other techniques in tandem with CLSM-FISH [40]. Other technical limitations of CLSM include the need for fluorescence staining of the biofilm molecules and the inability to render biofilms with a thickness of more than 150 µm. Novel technologies and sophisticated approaches have empowered CLSM to overcome some of these shortcomings.

Transmission electron microscopy (TEM) and scanning electron microscopy (SEM) are the two subcategories of electron microscopy (EM), both of which have been used by researchers recently for the three-dimensional analysis of biofilm structures. Electron microscopy functions based on the emission of a focused beam of electrons on the surface of the specimens and producing images based on the interactions of the electrons with specimen atoms. Rajeb et al. [41] employed SEM to illustrate the formation and expansion of microbial biofilm in near proximity of sand grains packed in a lab-scale wastewater treatment percolation cylinder. Baum et al. [42] conducted a study of *Pseudomonas fluorescens* isolated from the natural soil environment to find an estimation of the biofilm’s chemical composition, using light microscopy together with CLSM, TEM, and SEM. In a creative method to monitor biofilm in situ by using an optical microscope, de Carvalho et al. [25] developed a method based on the linear relationship that the intensity of a pixel of biofilm has in the *x*-*y* plane and the number of cells in the *z*-direction. They tried to investigate the biofilm 3D structure with brightfield transmitted and fluorescence light with an optical microscope. While unable to create 3D images, brightfield light transmittance is also the principle behind determining biomass concentration in spectroscopy methods.

Environmental scanning electron microscopy (ESEM) is the modified version of SEM that eliminates the occurrence of mass loss and shrinkage owing to sample preparation procedures required for classical SEM [43]. ESEM benefits from a reduced sample-preparation time compared to SEM, thus making it far more capable for in situ usage. However, factors such as beam radiation may adversely influence the integrity and viability conditions, even though ESEM benefits from reduced or variable pressure chambers instead of the high vacuum pressures used in SEM [44]. Cabala and Teper [45] recommended ESEM as an efficient method in examining the crystallization of minerals in the rhizosphere environment. With the help of this method, they were able to gain an understanding of the regional metalliferous pollution of a zinc–lead mining area in southern Poland by studying the metal depositions in the rhizosphere vicinity of regional plants. Low image resolution was reported due to the lack of conductivity in hydrated samples [46,47].

### 2.2. Piezoelectric

**Figure 2:**
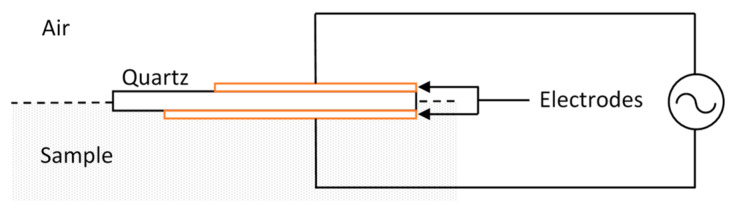
Simple schematic describing the basics of piezoelectric sensors.

Microbial monitoring using piezoelectric systems are based on the effect arising from bacterial mass formation on the surface of electrodes. It involves transmitting an alternating current (AC) to the sample through metal electrodes formed on glass (quartz crystal) substrates to induce the oscillation of the electrodes (Figure 2). The formation of a mass of biofilm on the surface of the electrode increases the oscillating mass and, therefore, causes a decrease in the measured output frequency that can be monitored as a measure of biofilm formation compared to when no biofilm exists on the electrode surface. Quartz crystal microbalance (QCM) is one of the piezoelectric devices that uses the adhesion force of microbial biofilms [47]. Several researchers, such as Nivens et al. [48], have developed QCM piezoelectric devices that are capable of monitoring biofilms.

### 2.3. Fiber-Optics

**Figure 3:**
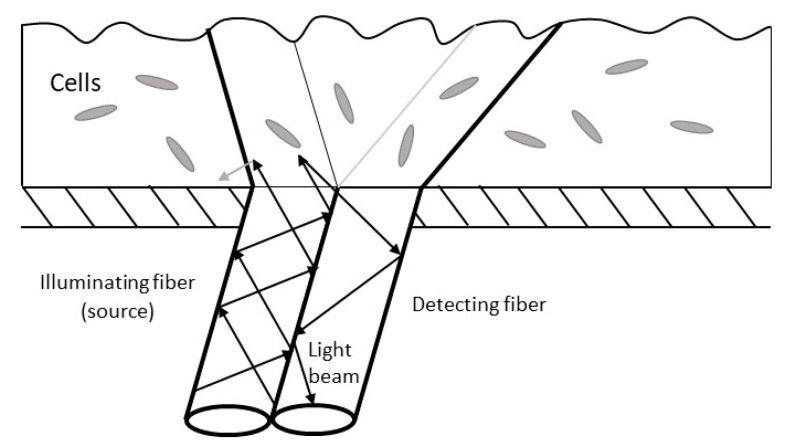
Basic premise behind a fiber-optic sensor.

Fiber-optics are an optical-based sensor in which the differences in the turbidity, light scattering, light absorption, reflectance, refractive index, fluorescence, bioluminescence, and surface resonance resulting from the corresponding interaction between the biofilm and the light source, are sensed by the fiber-optic [36,49] (see Figure 3). Turbidity and surface sensitivity induced by the biofilms are the two measurands that are most commonly measured by optical sensors for biofilm monitoring. Surface-sensitive sensors are developed to improve the signal-to-noise ratio or biofilm-sensing efficiency and monolayers of biofilm by the means of evanescent field sensors [49]. About two decades ago, researchers proposed a fiber-optic sensor (FOS) that was capable of monitoring the deposition of substances on the tip of the fiber [50]. The need for biofouling-prevention sensors motivated researchers to craft an FOS for monitoring the biofilm in the groundwater piping system feeding into a brewery [51]. The sensor, which had a quartz polymer optical fiber head of 0.2 mm in diameter, successfully tracked biofilm growth after reaching 10^5^ cells/cm^2^ up to 10^10^ cells/cm^2^. Fischer et al. [52] designed an optical fiber sensor that is suited for use in natural aquatic environments. The device works based on the intrinsic fluorescence properties of the biofilm protein. The working basis of this device is to first back-illuminate the biofilm that forms on a transparent substrate using an ultraviolet–light-emitting diode (UV-LED) and then collect fluorescence by employing numerous optical fiber sensors. The intrinsic fluorescence of the amino acid tryptophan is excited at a specific wavelength and is detected at a different wavelength by using a numerically optimized sensor head that has a UV-LED light source and optical fiber bundles that collect fluorescence light. This system was proposed to be used in the Baltic Sea to monitor biofilm over a period of twenty-one days. Modeling and simulation of the system was performed prior to the experimentations in order to have the optimized design [52]. The researchers reported a detection limit of 4 × 10^3^ cells/cm^2^.

### 2.4. Thermometric Biofilm Sensing

**Figure 4:**
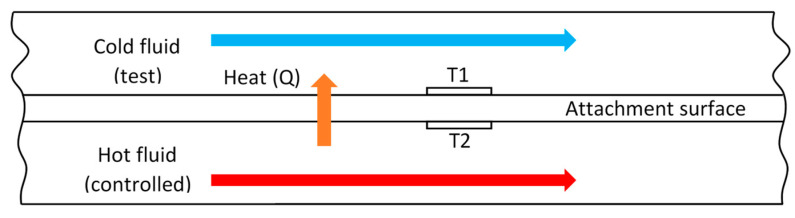
Basic setup behind a thermometric sensor (thermocouple 1 is shown as T1, and below it is thermocouple 2, indicated as T2).

In the field of biosensing, thermometric transducers measure the amount of heat induced by bacteria presence with a thermistor that is sensitive to heat [53] (see Figure 4). Like piezoelectric sensing of biofilm, the premise behind the thermometric method is the intensification or reduction of the measurand caused by the attachment of bacterial biofilm on the controlled surface. The deposition of bacterial biofilm on the surface causes an additional resistance against the flux of heat between known temperatures of both sides of the surface [33]. The differential flux of heat induced by the adhesion of biofilm on the surface of the channel walls is measured and interpreted as the thickness of the adherent biofilm. Reyes-Romero et al. [54] designed a novel thermometric sensor that utilized AC signals in creating oscillating heat through the heater that is in contact with the surface upon which the biofilm grows. A temperature probe was also placed in contact with the surface to measure the dynamic thermal fluctuations of the growth medium as a measure of biofilm formation. Even though they obtained varying signals as a result of bacteria proliferation, there was a lack of quantitative assessment or verification of the capability of the system. Moreover, no specific measure of the effectiveness of the device in terms of biofilm thickness or bacterial population was reported.

### 2.5. Electrochemical Sensing of Biofilm

**Figure 5:**
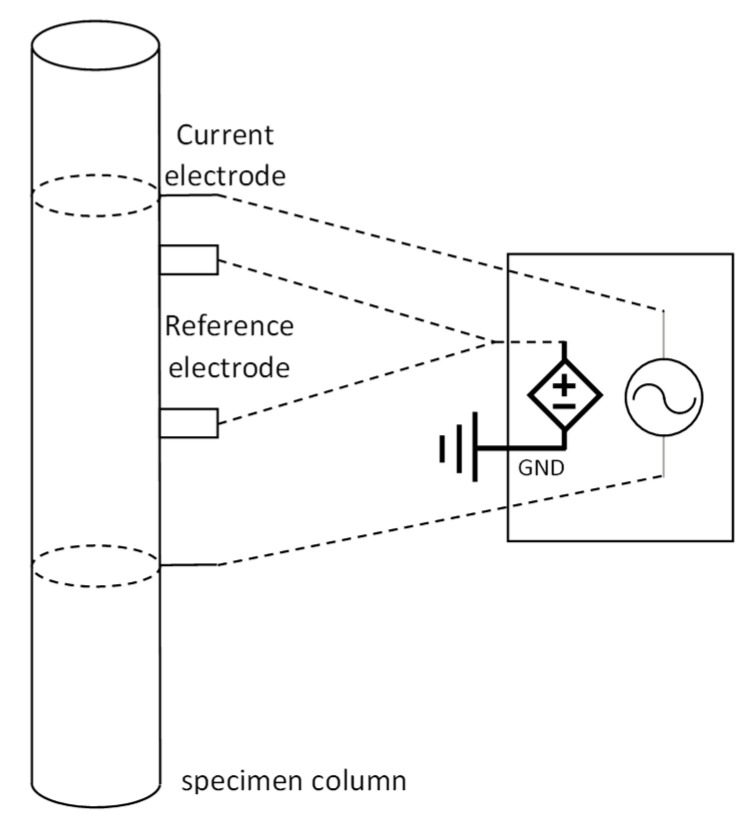
Basis of an electrochemical sensing method.

The metabolism of bacteria occurs through a series of biochemical reactions such as converting large molecules into smaller ones and releasing the energy, as well as utilizing organic and inorganic compounds for their maintenance and growth [55]. According to Brosel-Oliu et al. [56], the conversion process of large substrates into smaller charged and ionic metabolites leads to a change in the ionic composition of the cells that can be measured via electrochemical methods as a measure of bacterial metabolism or growth. This has likely contributed to electrochemical biosensing being the focus of much of the biofilm-related sensing research and development in recent years. Rapid responses, relatively easy-to-interpret data collection, and high sensitivity are just a few of the reported strengths of this method in comparison with other methods. There are multiple ways of obtaining electrical signals from a specimen of biofilm. Electrochemical biosensing works based on the idea of tracking the alterations of the output signal with respect to a controlled input (see Figure 5). Amperometric, potentiometric, conductometric, and impedimetric biosensing are the different classes of electrochemical sensing of bacterial presence.

Amperometric biosensors function based on the electric current generated by the oxidation–reduction reaction of species in contact with the surface of the working electrodes, while keeping the reference electrode at a fixed potential [57]. Amperometry is widely used in environmental monitoring, especially for the determination of biochemical oxygen demand (BOD) in water samples. However, the downside is the need for the available oxidizing and reducing agents in the circuit which are usually not present when dealing with biofilms. Conductometry is a fast and relatively simple method that is used not only in the field of biosensing but also in many industrial applications. It is based on the electrical conductivity of the analyte solution that is basically the opposite value of its electrical resistance. Despite its rapidity and ease of use, conductometry is not a selective method for biofilms, in that the changes in the analyte in terms of organic and/or inorganic content that can cause alterations in the electrical conductivity might also be interpreted as biofilm emergence or removal.

Impedimetric biosensing has gained significant interest in fields related to studying biological species in general and bacterial biofilm specifically. This has led to the advent of the field of impedance microbiology (IM), which is simply the application of impedance spectroscopy on microbiological species. This method involves measuring the impedimetric features of bacterial samples as a measure of their growth and availability in the system. Electrical impedance is the resistance of the circuit against the flow of current when a certain voltage is applied. Impedance, however, is not only the resistance of a resistor in the circuit but also the resistance from any capacitance and inductance features available in the system, if there are any. This stems from the fact that, in certain conditions, other than resistors, capacitors and inductors can display resistance against the flow of the current. Therefore, resistance, capacitance, and inductance could create impedance that includes at least one, and up to all of these, three features at the same time. If the voltage applied is through a direct current (DC), an inductor of the system behaves like a normal wire and does not display any inductive resistance, and a capacitor in the system acts like an open circuit, such that the resistance is infinite. However, when the voltage applied to the circuit is through an alternating current, any resistor, capacitor, and inductor available in the system will display some measurable impedance.

Living cells are composed of a closed, insulating membrane that is filled with liquid plasma and shows dielectric properties [33]. Such a structure allows them to behave like electrical capacitors that are built to store electrical charge when an electric current is applied [58]. When cells are exposed to an electric field, the ions available in plasma tend to move towards the cell membrane, creating a movement of ions which also induces a change in the electric field. These changes can be measured by controlled signals transduced from the signal source through the electrodes in contact with the biofilm. Moreover, extracellular electron transport (EET), which is a well-known process utilized by microorganisms for transporting electrons between the intracellular metabolic processes and the extracellular electron donors or acceptors, generates alterations in the electron transport and, therefore, resistance of the system under measurement [59]. Hence, other than capacitance, microbiota activity and proliferation are expected to be measurable through the tracking of the resistance. There is, however, no practical or theoretical evidence of microbial activity involving the creation of inductive properties in the system. Therefore, the impedimetric sensing of biofilms involves the measurement of resistance and capacitance of the biological samples of interest via an AC sinusoidal excitement. In this case, the impedance involves a contribution from the resistance (the real component of impedance) and the reactance (the imaginary portion), with the latter being related to the storage of charge in a conductive medium that comes in two forms: capacitance and inductance [60]. As discussed above and due to the unavailability of inductance in microbial samples, reactance is interpreted as capacitance.

There have been numerous research works based on the idea of impedance microbiology. From food safety monitoring [61] to point-of-care diagnosis of clinical diseases [62], impedance microbiology has been the main premise behind the monitoring systems developed. While there are no documented reports of direct impedance sensing of biofilm-colonizing plant roots, there have been multiple research works focused on the biofilm formed in subsurface environments. Frequency-domain-induced polarization (FDIP), which is vastly used in mining, hydrogeological, oil–gas, and environmental [63] studies for the assessment of geologic media, is conducted based on polarizing the media using AC signals, similar to impedance spectroscopy. In some instances, such as the work performed by Revil et al. [64] on brine-saturated clayey soils, the word “induced polarization” is used as an alternative name for complex conductivity, which is just an alternative name for AC impedance spectroscopy. Longo et al. [34] developed an impedance-based sensor applied at the microwave range to detect the initiation of biofilm in cases where low detection levels are desired. The sensor used an open-ended coaxial probe in the microwave range, which was shown to be highly sensitive in the early stages of biofilm growth, using *Vibrio natriegens* and *P. aeruginosa*. Davis et al. [65] studied the dynamic changes of impedimetric properties of biostimulated sand-packed columns. They crafted two 30 cm long experimental columns filled with sand grains and placed two electrode coils 16 cm away from each other along the length of the columns for current injection. The columns were fed with different fluids circulated throughout the entire length of the columns, using a peristaltic pump. This was performed to encourage different biological and physicochemical environments and compare the different impedimetric properties. Using a dynamic signal analyzer, AC signals would be transduced to the electrodes, and the conductivity magnitude (reverse of impedance) and the phase shift were measured over 60 days, and the real and imaginary components of conductivity (reverse of impedance) in columns were calculated. ESEM imaging was also conducted to confirm the microbial growth in any of the columns. They observed a rise in the imaginary conductivity that was inferred to be due to the aggregation of the bacterial cells and/or the adherence of biofilm to the sand grains. The ESEM images of sand grains from the unstimulated column did not lead to a corresponding relationship with the small impedimetric changes in this column. The authors concluded that the imaginary component of impedance can be interpreted as complex conductivity or capacitive impedance and can be utilized as a solid indicator of microbial growth and formation. Davis et al. [65] also proposed that this method can be used for the investigation of the integrity of biofilms in the process of contaminant remediation.

## 3. Discussion

An ideal biofilm-monitoring system for a rain garden in the field would be a system that can function non-destructively, continuously, and without impacting the microbial community. It should have fast, accurate online feedback acquisition. Researchers have been developing systems to achieve bacterial biofilm monitoring that fall into several categories, including microscopical [66], piezoelectric [48], fiber-optic [67], electrochemical [68], and thermometric [52]. This classification is based on the transducing element utilized in each method. The following discusses the general advantages and disadvantages of each method reviewed above, as well as their potential for use in a field application measuring bioremediation in a rain garden in situ.

### 3.1. Microscopical

Microscopy is the traditional method used for monitoring biofilm [69], and it remains the only direct method for observing and imaging bacterial biofilms. It is considered accurate and, thus, is often used to calibrate/support other indirect methods of measuring biofilm characteristics. Microscopical methods are typically labor-intensive and time-consuming, often requiring lengthy procedures and implementation by trained personnel, as well as the removal of the samples from their own host environment. This removal is usually achieved by exposing test surfaces called coupons inserted in situ that are removed from the environment of interest after a certain time and evaluated in the laboratory [50]. This potentially alters and damages the biofilm in the bioretention cell, and it is impossible for results to be reported in real time or online; thus, the results are not representative of the metabolism of bacterial biofilms in their environment. Being able to monitor biofilm inoculation in real time allows for the tracking of possible changes in the system as they occur. This is beneficial both when the static growth in the host environment is of interest and when the effect of certain chemical agents on the formation and proliferation of biofilm is of interest [53]—both of which could lead to crucial improvements in the engineering of the bioretention cells and therefore, the efficacy of water-quality improvements.

Microscopical methods could be used in combination with each other to fill the gaps and constraints of one another. ESEM, for example, can be used jointly with the CLSM-FISH method to provide an estimation of the volume containing the total cell counts (determined by CLSM-FISH) per substratum [43]. However, these techniques, although powerful, genuinely require large lab spaces and roofed areas. CLSM systems and microscopic techniques, in general, lack portability and applicability for field use, which is a requirement for monitoring rhizosphere biofilms. Moreover, the types of equipment used for three-dimensional microscopy are in the category of highly expensive facilities and always require trained personnel to operate them. Furthermore, extensive sample preparation and limited bacteria quantification are the other disadvantages of these techniques. One of the disadvantages of the SEM microscopical method is the need for a high vacuum for the assessment of biological samples. This not only requires the sample to be in the solid phase but could create artefacts on the structure of the biofilm [70]. The high-vacuum-pressure environment is used between the electron optic column and the sample chamber to prevent the scattering of electrons on its path to the specimen [71]. Microscopic methods are not an option for the rapid, portable, or online monitoring of biofilms in the field. Other than these factors, it is the best system for assessing a bacteria population over a wide range of concentrations. Therefore, microscopical techniques are best suited for the calibration and verification of alternative methods.

### 3.2. Piezoelectric

These devices provide the means for real-time and non-destructive biomass monitoring. The main downside of this technique, however, is the necessity of biofilm adhesion onto the surface of the electrodes. This is a limitation of use, especially for bacteria in aqueous media—where existing unattached bacteria in the sample are of interest. This could also apply to biofilms that are loosely attached [47]. The formation of biofilms occurs in a three-dimensional space where the extracellular matrix forms as a result of binding and aggregation; however, the quartz crystal microbalance devices may not be capable of measuring the cells and matrices formed on top of the bound layer. Therefore, the lack of depth resolution can be regarded as one of the important drawbacks of piezoelectric systems. It is also reported that the detection of biofilm is feasible when the biofilm is thin and does not have high bacterial concentrations. Nivens et al. [48] reported a detection limit of 3 × 10^5^ cells·cm^−2^ for *Pseudomonas cepacian* bacteria with this method. In addition, the environmental pressure and temperature that the crystal was exposed to affected the oscillation frequency. This indicates the need to use QCM in temperature- and pressure-controlled environments or to use techniques to compensate for such effects. This effectively eliminates their use in the application of interest in this work. Other types of piezoelectric sensors have been developed to detect different kinds of vibrations, but there is no evidence of their employment in the realm of biofilm monitoring [33].

### 3.3. Fiber-Optic Sensors

The main benefits of using fiber-optic sensors are the low power consumption and low cost. Another advantage is the fact that LEDs are able to stop and start illumination in a few milliseconds, thus empowering the system to emit reproducible light intensities. Ming et al. [72] designed an experimental setup of a fiber-optic biochemical gas senor that was capable of “sniffing” the formaldehyde (called FA) released in the process of formaldehyde detoxification using foliage plants. Using UV-LED illumination (λ = 340 nm) of nicotinamide adenine dinucleotide (NADH) (a product of FA dehydrogenase reaction), they reported the successful monitoring of FA concentrations as low as 2.5 ppb and as high as up to 100 ppb. Fischer et al.’s [52] system was reported to have a detection limit of 4 × 10^3^ cells/cm^2^, which is also low. However, to date, there is no report of the successful use of fiber-optic sensing in the subsurface and soil environments. Moreover, the geologic environments are genuinely opaque media that contain abiotic and grain-like substances that could potentially disrupt the process of illumination and detection in such sensors. Other disadvantages include the fact that optical fibers are not suitable for the monitoring of thicker biofilms [33]. This suggests they are not a primary method for measuring plant-associated biofilms but may be used as a reliable method when measuring the degassing of biofilms. This could serve as a surrogate measure of biomass activity in situ.

### 3.4. Thermometric

In general, a disadvantage of thermometric sensors for use in the field is the additional instrumentation required for the sole purpose of keeping the environmental temperatures optimal for biofilm formation. This optimal temperature is not necessarily constant but less dynamic than natural changes to allow for the biofilm formation heat effect to reach a sensible signal-to-noise-ratio. According to Janknecht and Melo [33], this technique does not have enough sensitivity to monitor the initial attachment of bacteria and probably cannot detect the adhesion first layer(s) of biofilm. However, it should be noted that the point of interest for measuring biofilm activity in a rain garden is not necessarily the initial attachment, or even the growth stages, but predominantly in the steady, mature phases of biofilm activity.

### 3.5. Electrochemical

Potentiometric methods that are based on the measurement of the potential differences between a working electrode and a reference electrode separated by a membrane suffer from the same weaknesses as those of amperometric systems when it comes to measuring biofilms. In addition, potentiometry requires a very stable and accurate reference electrode that is genuinely a challenge to maintain [73]. Therefore, potentiometric and amperometric forms of electrochemical techniques do not seem to be applicable for use in biofilm monitoring in rain gardens. This is especially true in subsurface environments where the space is limited and the use of potentiometric electrodes is nearly impossible, as the miniaturization of such electrodes is cumbersome.

According to the literature review, impedance-based methods are the most promising electrochemical methods for monitoring biofilms. Frequency-domain-induced polarization, also known as complex conductivity or impedance spectroscopy, has the potential to be a suitable monitoring method for geologic media. A major advantage of impedance spectroscopy is the minimal sample preparation. This means that continuous monitoring is possible without having to physically exploit samples and interrupt the host environment [74]. Recent advances have shown the potential of frequency-domain-induced polarization to provide plant-root structure and characterization measurements based on the biogeophysical responses acquired from plant root cells [75]. The advances in technologies such nanofabrication and lithography have made the miniaturization of electrodes used in impedance biosensing possible to the extent where most of the bacteria-related impedance sensing is performed via microscale electrodes known as Inter-Digitated Arrays (IDAs) or Inter-Digitated Micro-Electrodes (IDEs). Other than miniaturization, rapid advancements in the means of signal transduction, computing software, and device automation and control have contributed greatly to the betterment of biosensing technology [36]. IM has the advantages of online monitoring, flexibility, and customizability based on the application, high sensitivity, extensive evidence of field use, and potential for low-cost and portable implementation. One major downside to impedance spectroscopy, however, is the extremely long testing period—23 days under stimulated conditions. This is due to the fact that the system is only sensitive to substantial biological changes and a vast amount of biofilm formation is needed for the system to react. In other words, the signal-to-noise ratio or biofilm-monitoring efficiency is considered low. To overcome this shortcoming, miniaturization of the system is required to allow for higher sensitivities to biofilm growth and availability. Paredes et al. [76] also suggested that the smaller the area of the electrodes, the less biological material is required to observe a measured influence.

## 4. Conclusions

The ideal monitoring system of biofilm in situ in rain gardens (bioretention cells) should include the following functional requirements: operation in real-time, non-invasive, non-destructive, online, quantitative, portable, autonomous, sufficiently long testing uptime and signal acquisition, large substrate surfaces, and affordable tracking. Despite vast efforts and the advances in the field of biosensor development, to the authors’ knowledge, there is still no sensor that is commercially available and can be used at an industrial scale and for field use in rain gardens to determine biomass activity leading to bioremediation. This review paper examined the possible methods for measuring biofilm characteristics as an indicator for bioremediation in rain gardens. Microscopical methods form the traditional method for the direct and accurate measurement of biomass, but they lack the portability and quick analysis required in the field. Thermal and piezoelectric sensors typically require the formation of biofilm layers in the order of tens of micrometers to show a response. These are not options for the early detection of bacterial biofilms that is highly crucial in many different applications [77]. In the context of bioretention cells, interest is generally at the mature biofilm stage when bioremediation and stormwater treatment are at their maximum. It does suggest, however, that their use is limited to a period in which the rain garden is functioning optimally and cannot provide a reliable indication of any other stage of biofilm formation and bioremediation function. The low detection limits of the optical sensors and their functional variations related to the environment do not make them dependable for use in geologic media such as soil. Impedance biosensing, which is an electrochemical sensing method offers flexibility, customizability, high sensitivity, and supporting evidence in field use. It also has the potential for low-cost and portable implementation.

To reach the objective of developing a portable, low-cost, effective tool for biofilm characterization in a rain garden, lab-scale implementation and testing is certainly required during development. There are a few commercially available systems that are compatible with lab-scale use, but a lack of guidance for verification methods, sensitivity, and detection thresholds suggests the need for further implementation and development of a well-calibrated field-scale biosensing system. Moreover, a system is required for researchers to be able to study the effect of environmental conditions on the growth and metabolism of different biofilm-forming microorganisms to better engineer the bioretention cells. In addition, field use without lab-scale and pilot-scale verification could bring serious uncertainties in the device.

Impedance biosensing is recommended as the main method behind developing a sensor for use in monitoring environmental biofilm in rain gardens, but with microscopic and optical density methods used for verification and calibration of the sensor. Furthermore, rather than trying to execute the impedance experimentation on the biofilm around plant roots in uncontrolled environments, bacterial samples in known growth media can be used to help calibrate the device. The parameters that can be measured using such a system would have to characterize the relationship between the electrical capacitance/resistance and biofilm growth.

## Data Availability

No new data were created in this work.
